# Flexible control and trajectory planning of medical two-arm surgical robot

**DOI:** 10.3389/fnbot.2024.1451055

**Published:** 2024-08-27

**Authors:** Yanchun Xie, Xue Zhao, Yang Jiang, Yao Wu, Hailong Yu

**Affiliations:** ^1^Department of Orthopedics, General Hospital of Northern Theater Command, Shenyang, China; ^2^Daniel L. Goodwin College of Business, Benedictine University, Chicago, IL, United States; ^3^Faculty of Robot Science and Engineering, Northeastern University, Shenyang, China

**Keywords:** medical two-arm robot, momentum observer, motion control, trajectory planning, FKP

## Abstract

This paper introduces the flexible control and trajectory planning medical two-arm surgical robots, and employs effective collision detection methods to ensure the safety and precision during tasks. Firstly, the DH method is employed to establish relative rotation matrices between coordinate systems, determining the relative relationships of each joint link. A neural network based on a multilayer perceptron is proposed to solve FKP problem in real time. Secondly, a universal interpolator based on Non-Uniform Rational B-Splines (NURBS) is developed, capable of handling any geometric shape to ensure smooth and flexible motion trajectories. Finally, we developed a generalized momentum observer to detect external collisions, eliminating the need for external sensors and thereby reducing mechanical complexity and cost. The experiments verify the effectiveness of the kinematics solution and trajectory planning, demonstrating that the improved momentum torque observer can significantly reduce system overshoot, enabling the two-arm surgical robot to perform precise and safe surgical tasks under algorithmic guidance.

## 1 Introduction

With the continuous development of medical technology and the progress of robot technology, more and more robots are applied in the medical industry (Zhong et al., 2019). The flexible control and trajectory planning of medical dual-arm collaborative robots are crucial technologies enabling these robots to perform various complex tasks in the medical field, such as surgical assistance (Wu et al., 2019) and rehabilitation training (Culmer et al., 2009). Flexible control (Chalhoub and Ulsoy, 1987) refers to the ability of the robot control system to adapt to external environments and task requirements. For example, in the medical surgery, the responsive implementation, real-time control systems are crucial. These systems are adept at detecting and adjusting to minute fluctuations within the patient internal environment, thereby ensuring both the precision and safety of surgical procedures. Additionally, these adaptable control systems make robots more skilled and flexible when they do difficult jobs. This is most noticeable when robots have to do tasks when they are working closely with people. Flexible control is often closely related to trajectory planning in medical dual-arm robots (Kojima and Kibe, 2001; Kojima, 2002).

Trajectory planning refers to determining the robot motion trajectory in the workspace to enable it to accomplish specified tasks (Asada et al., 1990). In medical dual-arm robots, trajectory planning must consider factors such as anatomical structures of the surgical area, surgical objectives, and safety to ensure that the robot motion can reach the expected positions accurately and safely.

In the standard operational procedures for industrial robots, ensuring both the robot functionality and the staff safety are paramount. To achieve this, a physical barrier is commonly implemented. This barrier serves to segregate the robot from the surrounding area or to delineate the operational zones of both the staff and the robot. This strategic separation effectively prevents any direct physical contact between the staff and the robotic equipment. In the medical sector, however, there is a distinct preference for robots that can collaborate closely with human operators. This necessitates the capability of robots to execute a multitude of tasks securely within the intricate and demanding conditions of surgical settings. The robots must be designed to operate harmoniously with medical professionals, ensuring that they can contribute effectively to the high-precision requirements of healthcare without compromising safety. Collision protection of medical dual-arm collaborative robots is critical to ensuring robot safe operation when working with humans. This protection typically employs various technologies and methods:

1. Sensor technology (Zeng and Bone, 2013; Popov et al., 2017; Li et al., 2020): Sensors installed at various vital parts of the robot, such as arms and around the body, detect proximity or collisions. These sensors can be proximity, photoelectric, or pressure sensors, among others.
2. Vision systems (Mohammed et al., 2017): Vision systems using cameras or laser scanners are utilized to detect the surrounding environment and human positions and timely identify potential collision hazards.
3. Collision detection algorithms (Cao et al., 2019; Han et al., 2019): Software algorithms analyze data provided by sensors and vision systems to determine the presence of collision risks and take corresponding measures to avoid collisions.

Adding sensors to the above collision detection method will increase the cost of the robot system, while sensorless collision detection will use acceleration information, which will lead to the introduction of interference and error.

In the robot arm kinematics, the research approaches (Venanzi and Parenti-Castelli, 2005; Pandey and Zhang, 2012) for kinematics modeling and parameter identification have reached a high level of maturity. Rectifying the kinematics parameters based on the general error model can directly enhance the accuracy of the robot arm end position. However, the following drawbacks may emerge during the process: a significant amount of calculation, a long period for identification parameter iteration, which severely affects the motion velocity. During the medical robot operating process, it is challenging to employ sensors to detect and provide real-time feedback on the end pose. Nevertheless, the artificial neural network (ANN) boasts strong self-learning and adaptive capabilities (Aoyagi et al., 2012; Angelidis and Vosniakos, 2014). In recent years, ANN has been extensively utilized in domains such as system optimization and intelligent control, among which the BP (Back Propagation) neural network is a multi-layer feedforward network trained in accordance with the reverse propagation algorithm. Utilizing the algorithm based on the neural network to estimate the pose at the end of the manipulator can circumvent certain issues in the process of parameter calibration.

This study delves into neural network technology, analyzing the inverse kinematics problem (IKP) and forward kinematics problem (FKP). The current FKP solving strategy relies on iterative methods, which incur high computational costs and long processing times and cannot achieve optimal real time operations. This paper proposes a neural network utilizing an improved form of multilayer perceptron for backpropagation learning to enhance the accuracy of solving the mechanical arm forward kinematics problem to the desired level and achieve real-time solutions.

Furthermore, the study investigates the establishment of NURBS curve-related techniques. It integrates NURBS curves into trajectory interpolation of medical dual-arm collaborative robots capable of handling any geometric shape. Additionally, this paper introduces compensation and variable damping design into the collision detection method based on the second-order momentum torque observer to improve robot collision detection real-time accuracy.

## 2 Related work

**Figure 1:**
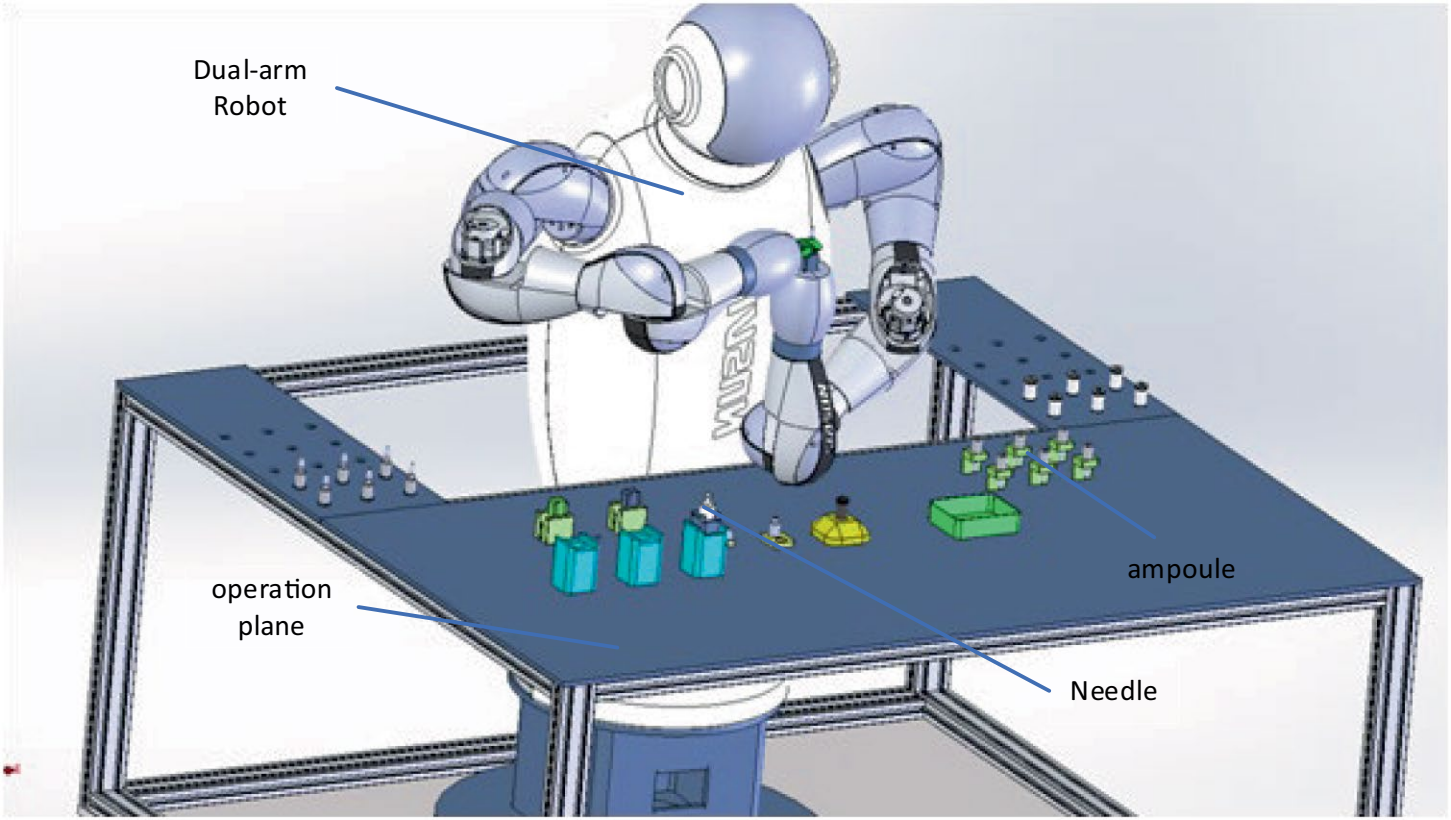
Medical dual-arm collaborative robot.

In the rapidly evolving medical industry landscape today, robotic automation has gradually entered the realm of medical systems. Traditional assembly processes of medical assistive devices are increasingly unable to meet the demands of modern medical production. Simultaneously, there is a growing demand for robots with high safety, stability, and flexibility to assist in collaborative operations during medical device-assisted drug dispensing. Robots can replace many tasks in medical drug dispensing. Manual solutions such as medication dispensing, assisting in guiding needles, and tumor resection using medical assistive devices must be updated. Medical dual-arm collaborative robots can perform complex drug dispensing processes, as depicted in Figure 1. The application of the medical dual-arm collaborative robot software platform in the medical assistive industry offers unparalleled advantages. These robots facilitate the drug dispensing process and enable collaboration between workers and robots. Integrating manual drug dispensing tasks with automated production addresses the labor-intensive operations in the medical industry. Moreover, the arms of these robots must possess the functionality for coordinated dual-arm operations to ensure synchronous and precise coordination and collision prevention.

### 2.1 Classification of robot interactive control

**Figure 2:**
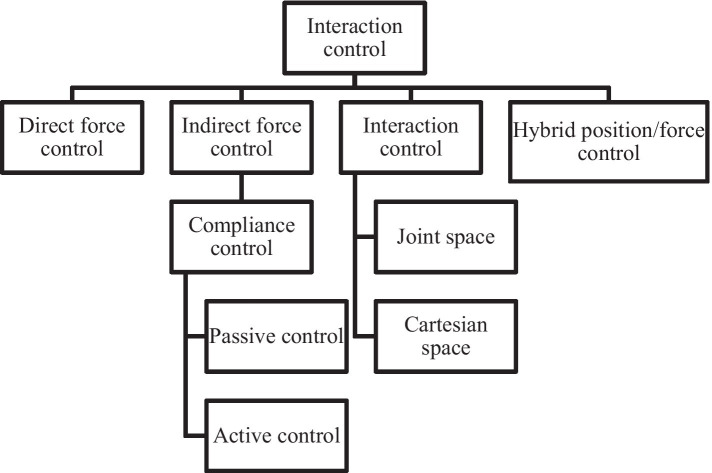
System composition of cleaning robot.

The classification of robot interaction control is illustrated in Figure 2. Common control methods (Niku, 2020) mainly include force/position hybrid control and impedance/admittance control. In several studies (Hogan, 1985; Anderson and Spong, 1988) on impedance control, a prescribed fixed passive impedance model is defined, and efforts are then focused on addressing challenges such as dealing with uncertainties. Research within this framework typically adopts either learning-based (Sharifi et al., 2021) or adaptive impedance control (Yu et al., 2019). However, assuming that a fixed impedance model is no longer sufficient to describe specific applications, such as explosive movements or Human-Robot Interaction (HRI). Therefore, adopting variable impedance control (Ikeura and Inooka, 1995) must be considered for robot interaction control. However, adjusting impedance parameters to provide optimal impedance characteristics is more effective in enhancing interaction performance, which is necessary for essential applications such as HRI. Physically informed HRI has been proposed in adaptive impedance kinematics learning. In this study (Dong and Ren, 2017), robots adapt their movements by learning tasks to predict the intentions of their partners. Optimal impedance adaptation in constrained motion HRI has been investigated (Sun et al., 2019). Continuous critic learning is employed for interaction control, followed by obtaining the desired impedance as the optimal implementation to meet the control objectives. However, adaptive control is not appropriate for the control system with a high real-time demand, and it is deficient in the capacity to handle nonlinear issues. Fuzzy control. The fuzzy processing of the control parameters might cause the reduction of precision and the deterioration of the dynamic quality of the force interaction control.

Li et al. (Li et al., 2016) explores the cooperative kinematic control of multiple manipulators through distributed recurrent neural networks and offers a feasible approach to extend the existing outcomes on individual manipulator control by using recurrent neural networks to the circumstance of the coordination of multiple manipulators. Jin et al. (Jin et al., 2022) analyses a collaborative control problem of redundant manipulators with time delays and proposes a time-delayed and distributed neural dynamics scheme. Yang et al. (Yang et al., 2023) proposes an Extended Kalman Filter-incorporated Residual Neural Network-based Calibration (ERC) model for kinematic calibration.

### 2.2 Arm mechanical structure

**Figure 3:**
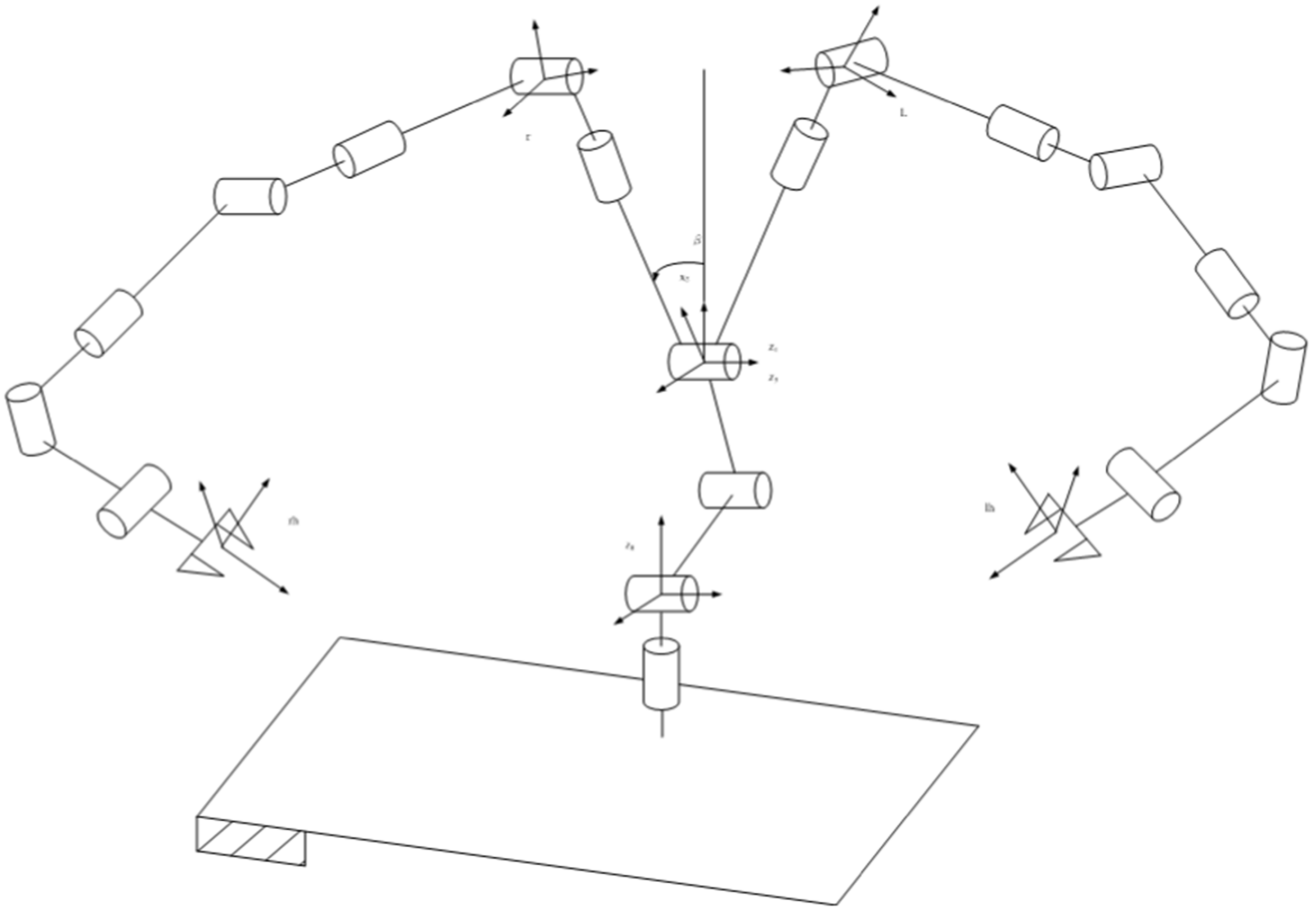
Coorinate system distribution.

As shown in Figure 3, the shoulder and elbow joints are designed with dual-degree mechanisms, differing only in motor arrangement. The motor achieves arm abduction through bevel gear transmission, while the offset motor achieves arm flexion and extension through spur gear transmission. The structural principles of the elbow joint are the same as those of the shoulder joint.

Peripheral components such as end effectors and dexterous hands are installed at the end, and backlash compensation is applied to the spur gear transmission system through an eccentric flange. The arm integrates driver boards for the elbow and wrist motors, facilitating later debugging and maintenance. Touch sensor modules can also be installed inside the arm to perceive human contact and respond with corresponding actions such as speech or facial expressions, enhancing interaction with humans.

## 3 Motion control and spline trajectory planning

### 3.1 Motion control of medical two-arm robot

#### 3.1.1 Kinematics forward solution

The humanoid two-arm robot has 14 degrees of freedom and can be analyzed for one hand and the other hand. The optional position of the base coordinate system is not unique. Establishing a coordinate system in the above way can facilitate the analysis of the geometric relationship between each joint value and the robot pose. Compared with the traditional D-H method, the homogeneous transformation matrix between the adjacent joints of the robot can be intuitively obtained during the forward solution process. At the same time, the calculation amount of inverse kinematics analysis can be reduced to a certain extent (Liu et al., 2021). This method is suitable for fast modeling in engineering, can simplify the derivation of kinematic relations, facilitate the acquisition of kinematic equations, and make the results more intuitive. Since the arm position is decoupled from the attitude, the first four joints and the wrist are calculated separately to facilitate the inverse solution. The robot is divided into four freedom degrees ( $\theta_{1},\theta_{2},\theta_{3},\theta_{4}$ ) at the lower part of the wrist and three degrees freedom ( $\theta_{5},\theta_{6},\theta_{7}$ ) at the end of the wrist joint. The forward kinematics is analyzed from the four joints at the base and three joints at the end. For the left hand, the transformation matrix from 1 to 7 can be obtained according to the spatial geometric relationship of each joint, as shown in Equation 1.


(1) $$T70=T40T74=\left[\begin{array}{cccc} ne0_{x} & oe0_{x} & ae0_{x} & pe0_{x}\\ ne0_{y} & oe0_{y} & ae0_{y} & pe0_{y}\\ ne0_{z} & oe0_{z} & ae0_{z} & pe0_{z}\\ 0 & 0 & 0 & 1 \end{array}\right]$$

The conclusion is as follows:


(2) $$\left\{\begin{array}{c} \begin{array}{c} {ne0}_{x}=-c7(s5(s4(c1s3+s1c2c3)+s1s2c4)\\ -c5(c1c3-s1c2s3))\;-s7(s6(c4(c1s3+s1c2c3)\\ -s1s2s4)+c6(s5(c1c3-s1c2s3)\\ +c5(s4(c1s3+s1c2c3)+s1s2c4))) \end{array}\\ \begin{array}{l} {ne0}_{y}=-s7(s6(c4(s1s3-c1c2c3)+c1s2s4)\\ +c6(s5(s1c3+c1c2s3)\\ +c5(s4(s1s3-c1c2e3)-c1s2c4)))\\ -c7(s5(s4(s1s3-c1c2c3)-c1s2c4)\\ -c5(s1c3+c1c2s3)) \end{array}\\ \begin{array}{l} {ne0}_{z}=s7(s6(c2s4+s2c3c4)-c6(c5(c2c4-s2c3s4)\\ +s2s3s5))-c7(s5(c2c4-s2c3s4)-s2s3c5) \end{array} \end{array}\right.$$

(3) $$\left\{\begin{array}{l} {oe0}_{x}=s6(s5(c1c3-s1c2s3)+c5(s4(c1s3\\ +s1c2c3)+s1s2c4))-c6(c4(c1s3\\ +s1c2c3)-s1s2s4)\\ {oe0}_{y}=s6(s5(s1c3+c1c2s3)+c5(s4(s1s3\\ -c1c2c3)-c1s2c4))-c6(c4(s1s3-c1c2c3)\\ +c1s2s4)\\ {oe0}_{z}=s6(c5(c2c4-s2c3s4)+s2s3s5)\\ +c6(c2s4+s2c3c4) \end{array}\right.$$

(4) $$\left\{\begin{array}{c} \begin{array}{l} {ae0}_{x}=c7(s6(c4(c1s3+s1c2c3)-s1s2s4)\\ +c6(s5(c1c3-s1c2s3)+c5(s4(c1s3+s1c2c3)\\ +s1s2c4)))-s7(s5(s4(c1s3+s1c2c3)+s1s2c4)\\ -c5(c1c3-s1c2s3)) \end{array}\\ \begin{array}{l} {ae0}_{y}=c7(s6(c4(s1s3-c1c2c3)+c1s2s4)\\ +c6(s5(s1c3+c1c2s3)+c5(s4(s1s3-c1c2c3)\\ -c1s2c4)))-s7(s5(s4(s1s3-c1c2c3)-c1s2c4)\\ -c5(s1c3+c1c2s3)) \end{array}\\ \begin{array}{l} {ae0}_{z}=-s7(s5(c2c4-s2c3s4)-s2s3c5)\\ -c7(s6(c2s4+s2c3c4)\\ -c6(c5(c2c4-s2c3s4)+s2s3s5)) \end{array} \end{array}\right.$$

(5) $$\left\{\begin{array}{c} \begin{array}{l} {pe0}_{x}=D_{2}s1s2+D_{3}s1s2-D_{4}(c4(c1s3+s1c2c3)\\ -s1s2s4) \end{array}\\ \begin{array}{l} {pe0}_{x}=-D_{4}(c4(s1s3-c1c2c3)+c1s2s4)\\ -D_{2}c1s2-D3c1s2 \end{array}\\ \begin{array}{l} {pe0}_{z}=D_{1}+D_{2}c2+D_{3}c2\\ +D_{4}(c2s4+s2c3c4) \end{array} \end{array}\right.$$

#### 3.1.2 Neural network solution

Installing additional sensors or transducers at the end of medical dual-arm collaborative robots to gather more information about the system state (robotic arm) may facilitate the rapid and convenient resolution of Forward Kinematics Problems (FKP). However, the additional cost of sensors renders it less than ideal. In iterative methods, kinematic problems are formalized, allowing them to be solved using any available numerical techniques. However, these numerical techniques are computationally intensive and cannot guarantee a solution.

The analytical approach of solving FKP based on neural networks is not limited to the specific structure of medical dual-arm collaborative robots; it can be extended to other types of six-axis medical dual-arm collaborative robots or generalized medical dual-arm collaborative robots. Neuron processing units sum modified signals and apply the result to a linear or nonlinear activation function. Subsequently, the generated signal or value is transmitted to output units. Inputs, weights, architecture, and thresholds are parameters neural network unit control.

Once the network designers determine the neural network architecture, weight values can be set through a training or learning process. In network training, the neural network free parameters (weights) adapt through a continuous stimulation process of the environment, embedding the network. Environmental stimuli are input–output data values obtained from different states of the environment. Free parameters are systematically updated during the training process to converge to optimal values. The learning rate controls the magnitude of free parameter updates. When to stop training depends on predefined conditions, such as reaching the maximum expected training time and the lowest error rate. The accuracy of neural network parameters and the amount of learning data are closely related to the learning process resembling that of the human brain. Inputs are the joint angles of the medical dual-arm collaborative robot, and the output of the neural network module is the position and posture of the robot end effector. First, nonlinear equations are established, as shown in Equation 6.


(6) $$\left\{\begin{array}{c} F_{1}=D_{2}s1s2+D_{3}s1s2-D_{4}\left(c4\left(c1s3+s1c2c3\right)-s1s2s4\right)-{pe0}_{x}\\ F_{2}=-D_{4}\left(c4\left(s1s3-c1c2c3\right)+c1s2s4\right)-D_{2}c1s2-D3c1s2-{pe0}_{y}\\ F_{3}=D_{1}+D_{2}c2+D_{3}c2+D_{4}\left(c2s4+s2c3c4\right)-{pe0}_{z} \end{array}\right.$$

**Figure 4:**
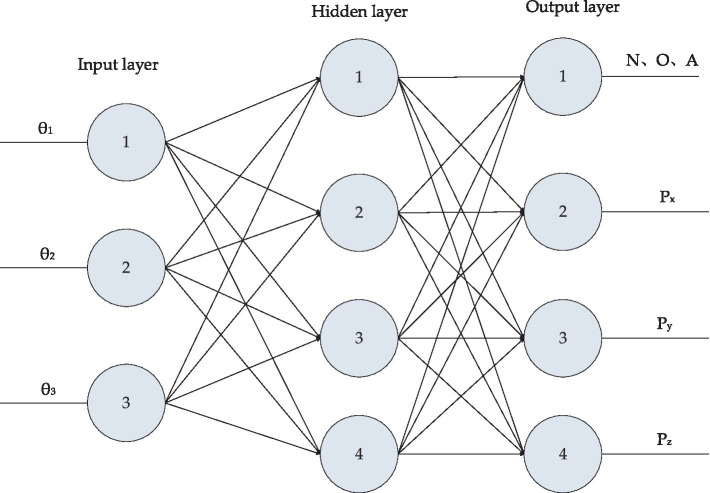
Three-layer feedforward neural network.

According to the aforementioned nonlinear equations, a three-layer BP neural network method is adopted to perform forward kinematics solution for medical dual-arm collaborative robot. As illustrated in Figure 4, the neural network topology consists of three layers, with the input layer comprising 3 neurons and the output layer containing 4 neurons. The nonlinear mapping of the robot forward kinematics can be converted into a linear mapping via Equation 7, where N samples are designated as $\left(P_{j}\text{,}\alpha_{j}\right)$ , with j=1,2,⋯,N . Here, $P_{j}$ represents the network input vector, serving as the j-th positional sample, and $\alpha_{j}$ represents the network output vector corresponding to the j-th motor angle.


(7) $$\{\begin{array}{c} h_{i}=f\left(w_{i}p_{j}+a_{1i}\right)\\ \alpha_{i}=f\left(w_{k}p_{i}+a_{2k}\right) \end{array}$$

Where: $w_{i}$ and $a_{1i}$ are the weights and thresholds of the node *i* of the hidden layer and the input layer; $w_{k}$ and $a_{2k}$ are the weights and thresholds between the output layer and the hidden layer.

From the experience gained through network training, it is understood that the number of training samples should be approximately 5 to 10 times the number of network weights. Once the sample set is determined, the initial step involves normalizing the input and output data of the neural network, ensuring that all data points are scaled to the range [−1, 1]. In the architecture of the BP network, the middle-hidden layer plays a pivotal role in the network performance, as it receives calculated results from the input layer and passes them to the output layer. Therefore, the activation function used in the hidden layer must be continuous and smooth to facilitate effective learning. Given its favorable properties and compliance with these criteria, the sigmoid function is chosen as the activation function for the hidden layer of the neural network. This choice is made to enhance the network ability to model complex relationships within the data.

**Figure 5:**
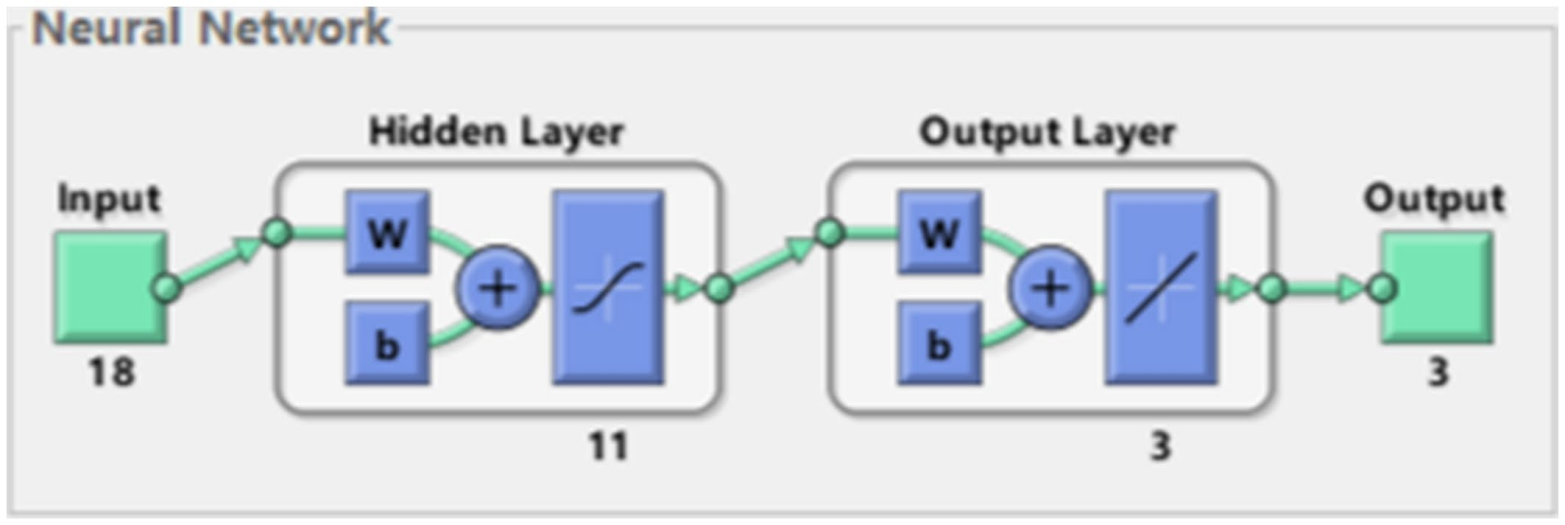
Network structure.

**Figure 6:**
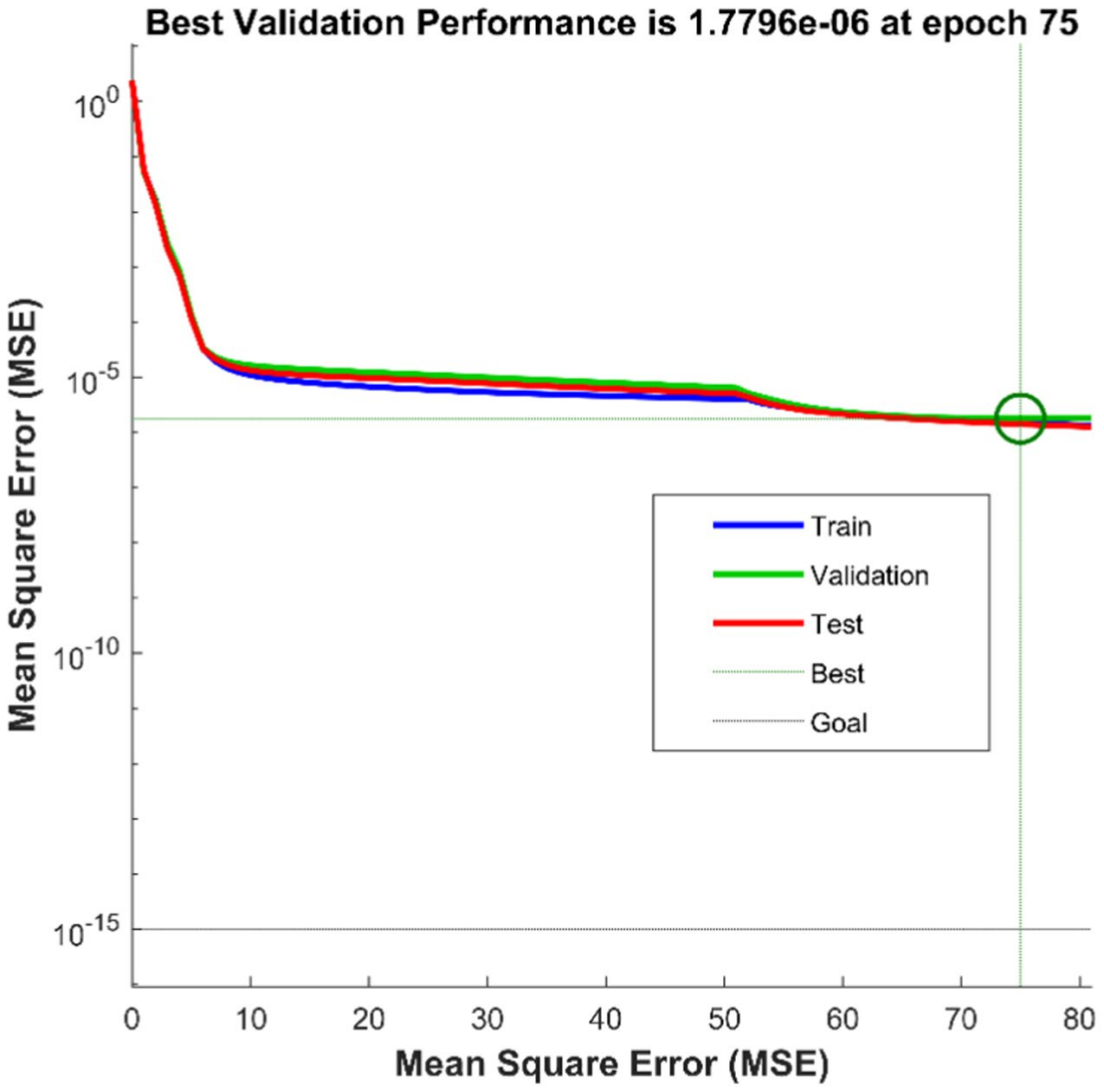
BP train result.

We take the position, velocity and acceleration of each joint as input to form an 18-dimensional input vector, and take the spatial coordinates of the end joints as output. The optimal number of hidden layers of the neural network can be obtained by empirical formula, which can be specifically seen in Algorithm 1. Using MSE as a metric, we can calculate that the best hidden layer node is 11. The whole network structure is shown in Figure 5. The training results are shown in Figure 6.

##### ALGORITHM 1. Optimal hidden neuron count for a backpropagation network

**Table 1:** 

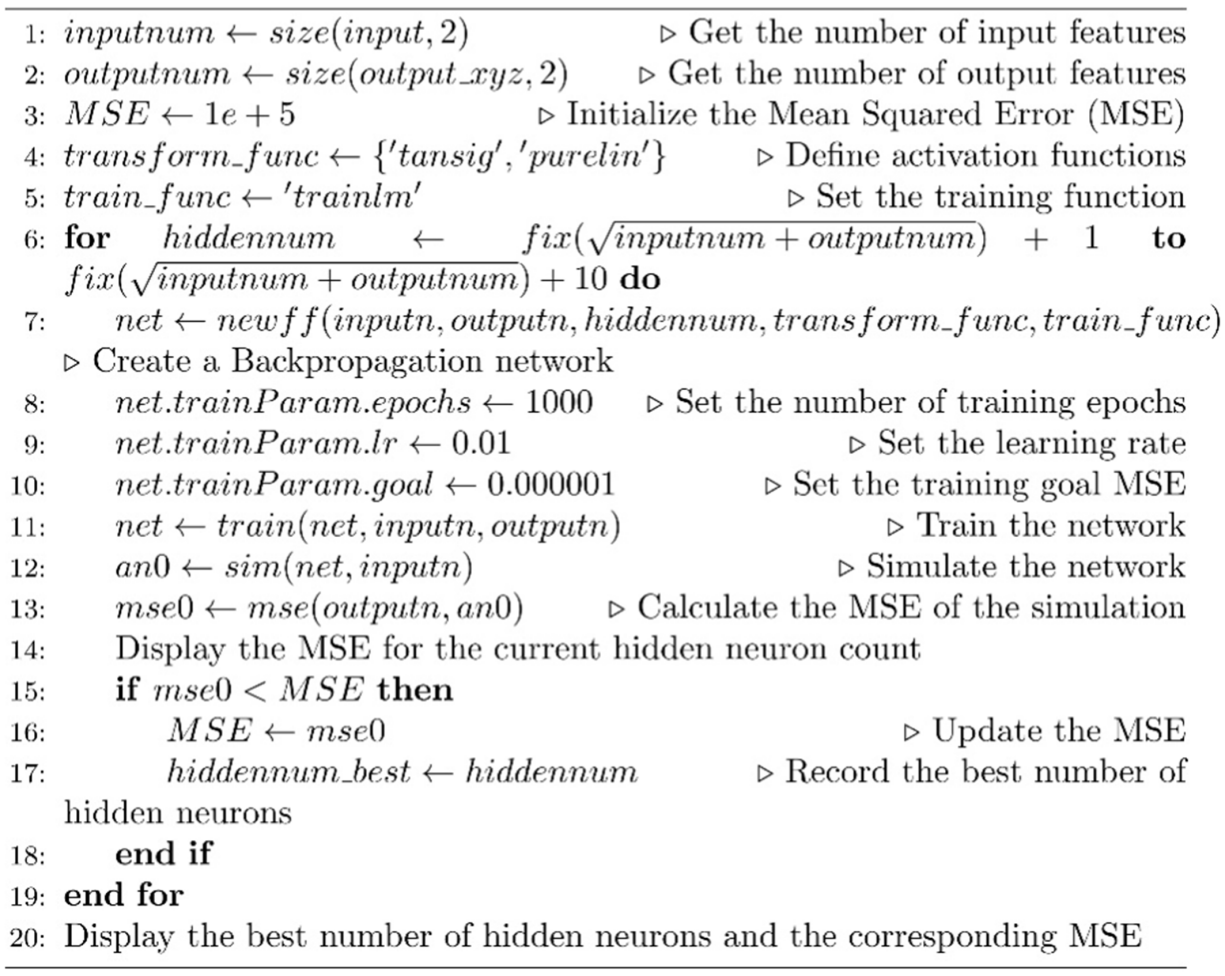

In the motion control process of the medical dual-arm collaborative robot, a large number of positions and joint angles are saved as samples. After training with these samples as inputs, the kinematic forward description from robot joint angles to robot end effector positions can be obtained. This allows the establishment of the nonlinear equations and weights to derive the forward kinematics of the medical dual-arm collaborative robot.

### 3.2 Trajectory planning with NURBS

NURBS (Non-Uniform Rational B-Splines) spline curves are a commonly used mathematical representation method in computer graphics and computer-aided design (CAD). A series of control points, weights, and knot vectors define them. NURBS curves are widely used in computer graphics and CAD because they can accurately represent various curve shapes and have good mathematical properties such as local control, smoothness, and adjustability. This makes them essential tools in design and modeling work.

**Figure 7:**
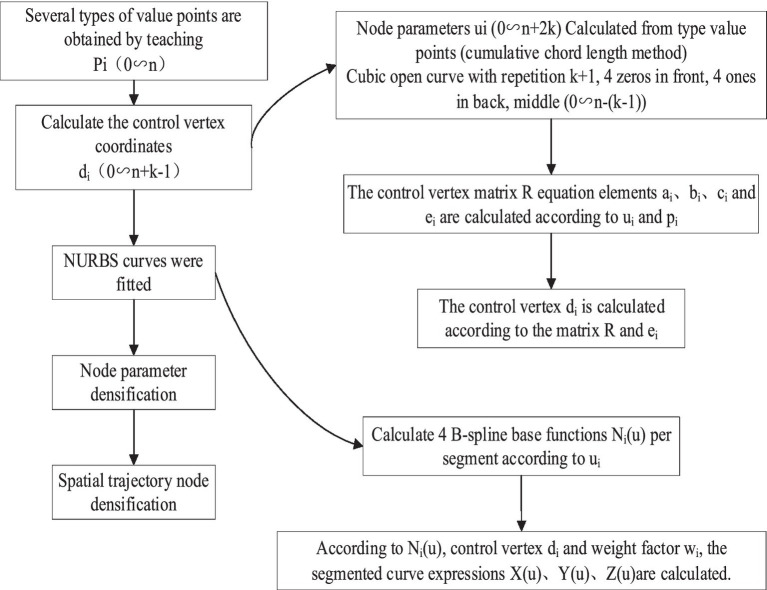
NURBS spline curve fitting proces.

The steps for NURBS spline curve planning involve specific algorithms to calculate the feed rate and sample points for each sampling interval, as this motion planning is implemented in real time. The NURBS spline curve fitting process is illustrated in Figure 7.


(8) $$\left\{\begin{array}{c} \Delta_{i}=u_{i+1}-u_{i}\\ \begin{array}{l} a_{i}=\frac{{\left(\Delta_{i+2}\right)}^{2}}{\Delta_{i}+\Delta_{i+1}+\Delta_{i+2}}\\ =\frac{{\left(u_{i+3}-u_{i+2}\right)}^{2}}{u_{i+3}-u_{i}},\;i=2,3,\cdots ,n \end{array}\\ \begin{array}{l} b_{i}=\frac{\Delta_{i+2}\left(\Delta_{i}+\Delta_{i+1}\right)}{\Delta_{i}+\Delta_{i+1}+\Delta_{i+2}}+\frac{\Delta_{i+1}\left(\Delta_{i+2}+\Delta_{i+3}\right)}{\Delta_{i+1}+\Delta_{i+2}+\Delta_{i+3}}\\ =\frac{\left(u_{i+3}-u_{i+2}\right)\left(u_{i+2}-u_{i}\right)}{u_{i+3}-u_{i}}\\ +\frac{\left(u_{i+2}-u_{i+1}\right)\left(u_{i+4}-u_{i+2}\right)}{u_{i+4}-u_{i+1}} \end{array}\\ c_{i}=\frac{{\left(\Delta_{i+1}\right)}^{2}}{\Delta_{i+1}+\Delta_{i+2}+\Delta_{i+3}}=\frac{{\left(u_{i+2}-u_{i+1}\right)}^{2}}{u_{i+4}-u_{i+1}}\\ e_{1}=p_{0}+\frac{\Delta_{3}}{3}p_{0}^{'}=p_{0}+\frac{u_{4}-u_{3}}{3}p_{0}^{'}\\ e_{n+1}=p_{n}-\frac{\Delta_{n+2}}{3}p_{n}^{'}=p_{n}-\frac{u_{n+3}-u_{n+2}}{3}p_{n}^{'}\\ e_{i}=\left(\Delta_{i+1}+\Delta_{i+2}\right)p_{i-1}=\left(u_{i+3}-u_{i+1}\right)p_{i-1}i=0,1,\cdots ,n \end{array}\right.$$

(1) Calculation process of node parameter $u_{i}$ (cumulative chord length method)

According to joint displacement time node sequence $\left\{p_{i}\text{,}t_{i}\right\}$ ; By definition, the corresponding node vector is $U=\left\{u_{0}\text{,}u_{1}\text{,}\cdots \text{,}u_{n+2k}\right\}$ ; The specific expression is shown in Equation 9.


(9) $$\{\begin{array}{c} u_{0}=u_{1}=\cdots u_{k}=0\\ u_{n+k}=u_{n+k+1}=\cdots u_{n+2k}=1 \end{array}$$

Formula $u_{i}$ calculated by cumulative chord length method:


(10) $$u_{i+3}=u_{i+2}+\frac{\left|p_{i}-p_{i-1}\right|}{\sum |p_{i}-p_{i-1}|}\text{​}i=1,2,\cdots ,n-1$$

(2) The calculation process of matrix R elements is shown in Equations 8 and 11.

(11) $$\left[\begin{array}{ccccc} 1 & \; & \; & \; & \;\\ a_{2} & b_{2} & c_{2} & \; & \;\\ \; & \ddots & \ddots & \ddots & \;\\ \; & \; & a_{n} & b_{n} & c_{n}\\ \; & \; & \; & \; & 1 \end{array}\right]\cdot \left[\begin{array}{c} d_{1}\\ d_{2}\\ \vdots \\ \vdots \\ d_{n+1} \end{array}\right]=\left[\begin{array}{c} e_{1}\\ e_{2}\\ \vdots \\ \vdots \\ e_{n+1} \end{array}\right]$$

(3) Control the calculation process of vertex $d_{i}$

The control vertex $dl_{1}$ is calculated according to the following Equation 12.


(12) $$\left[\begin{array}{c} d_{1}\\ d_{2}\\ \vdots \\ \vdots \\ d_{n+1} \end{array}\right]=inv\left(\left[\begin{array}{ccccc} 1 & \; & \; & \; & \;\\ a_{2} & b_{2} & c_{2} & \; & \;\\ \; & \ddots & \ddots & \ddots & \;\\ \; & \; & a_{n} & b_{n} & c_{n}\\ \; & \; & \; & \; & 1 \end{array}\right]\right)\cdot \left[\begin{array}{c} e_{1}\\ e_{2}\\ \vdots \\ \vdots \\ e_{n+1} \end{array}\right]$$

From the NURBS first and last points (control points), the Equation 13 can be obtained.


(13) $$\left[\begin{array}{c} dl_{1}\\ dl_{2}\\ \vdots \\ dl_{n+2}\\ dl_{n+3} \end{array}\right]=\left[\begin{array}{c} p_{0}\\ d_{1}\\ \vdots \\ d_{n+1}\\ p_{n} \end{array}\right]$$

(4) B spline basis function $Ni\left(u\right)$ has been calculated; The four basis functions of each segment B spline should be calculated according to the following formula, and the four basis functions $N_{j,3}\left(u\right)$ corresponding to $j=\left(i-3\text{,}i-2\text{,}i-1\text{,}i\right)$ should be calculated periodically for each segment, as shown in Equation 14.

(14) $$N_{j,3}\left(u\right)=\left\{\begin{array}{c} \frac{{\left(u_{i+1}-u\right)}^{3}}{\left(u_{i+1}-u_{i-2}\right)\left(u_{i+1}-u_{i-1}\right)\left(u_{i+1}-u_{i}\right)},\;j=i-3\\ \begin{array}{l} \frac{{\left(u_{i+1}-u\right)}^{2}\left(u-u_{i-2}\right)}{\left(u_{i+1}-u_{i-2}\right)\left(u_{i+1}-u_{i-1}\right)\left(u_{i+1}-u_{i}\right)}\\ +\frac{\left(u-u_{i-1}\right)\left(u_{i+1}-u\right)\left(u_{i+2}-u\right)}{\left(u_{i+1}-u_{i-1}\right)\left(u_{i+1}-u_{i}\right)\left(u_{i+2}-u_{i-1}\right)} \end{array}\\ +\frac{{\left(u_{i+2}-u\right)}^{2}\left(u-u_{i}\right)}{\left(u_{i+2}-u_{i-1}\right)\left(u_{i+2}-u_{i}\right)\left(u_{i+1}-u_{i}\right)},\;j=i-2\\ \begin{array}{l} \frac{{\left(u_{i-1}-u\right)}^{2}\left(u_{i+1}-u\right)}{\left(u_{i+2}-u_{i-1}\right)\left(u_{i+1}-u_{i-1}\right)\left(u_{i+1}-u_{i}\right)}\\ +\frac{\left(u-u_{i-1}\right)\left(u_{i+2}-u\right)\left(u-u_{i}\right)}{\left(u_{i+2}-u_{i-1}\right)\left(u_{i+2}-u_{i}\right)\left(u_{i+1}-u_{i}\right)} \end{array}\\ \begin{array}{l} +\frac{{\left(u_{i}-u\right)}^{2}\left(u_{i+3}-u\right)}{\left(u_{i+3}-u_{i}\right)\left(u_{i+2}-u_{i}\right)\left(u_{i+1}-u_{i}\right)},\\ j=i-1 \end{array}\\ \frac{{\left(u-u_{i}\right)}^{3}}{\left(u_{i+3}-u_{i}\right)\left(u_{i+2}-u_{i}\right)\left(u_{i+1}-u_{i}\right)},\;j=i \end{array}\right.$$

(5) Subsection curve expression calculation process

Calculate the expression of each B spline curve according to the following Equation 15, which needs to be calculated periodically for each segment:


(15) $$\{\begin{array}{c} Px\left(u\right)=\frac{\sum_{j=i-3}^{i}\omega_{j}px_{j}N_{j,3}\left(u\right)}{\sum_{j=i-3}^{i}\omega_{j}N_{j,3}\left(u\right)}\\ Py\left(u\right)=\frac{\sum_{j=i-3}^{i}\omega_{j}py_{j}N_{j,3}\left(u\right)}{\sum_{j=i-3}^{i}\omega_{j}N_{j,3}\left(u\right)}\\ Pz\left(u\right)=\frac{\sum_{j=i-3}^{i}\omega_{j}pz_{j}N_{j,3}\left(u\right)}{\sum_{j=i-3}^{i}\omega_{j}N_{j,3}\left(u\right)} \end{array}$$

At this stage, the B spline curve is fitted. Next, the curve data points need to be densified to enable the robot to walk to each densification point periodically. This densification is generally achieved through Taylor expansion.

### 3.3 Collision detection basics

Considering the influence of the external collision torque and the friction torque, the robot dynamic equation can be written as Equation 16. $q,\dot{q},\ddot{q}$ represent the robot joint angle, angular velocity and angular acceleration, respectively. Robot inertia matrix is *D*. Coriolis matrix is *C*. Gravitational term is *G*. The external impact is equivalent to the external torque of each joint is $\tau_{e}$ . Robot joint friction torque is $\tau_{f}$ . Robot motor drive torque is τ .


(16) $$D\left(q\right)\overset{••}{q}+C\left(q\text{,}\overset{•}{q}\right)\overset{•}{q}+G\left(q\right)+\tau_{e}+\tau_{f}=\tau$$

As can be seen from the Equation 16, solving $\tau_{e}\;$ requires the acceleration, but it will bring noise. So we design an observer based on generalized momentum. The robot generalized momentum is defined as Equation 17.


(17) $$P=D\left(q\right)\overset{•}{q}$$

We define an observation vector *r*. The torque gain matrix is *K_1_* and *K_2_*. $\hat{P}$ is the estimate of *P*. The second-order observer is shown in Equation 18.


(18) $$r=K_{1}\overset{t}{\underset{0}{\int }}\left(-K_{2}r+\left(\hat{P}-P\right)\right)dt$$

We optimize this second-order observer by introducing links such as Equation 19.


(19) $$u_{\text{f}}=\overset{t}{\underset{0}{\int }}\left(\tau -\tau_{f}+C^{\text{T}}\left(q\text{,}\overset{•}{q}\right)\overset{•}{q}-G\left(q\right)\right)dt-P$$

Finally, the resulting observer is shown in Equation 20.


(20) $$r=K_{1}\overset{t}{\underset{0}{\int }}\left(-K_{2}r+\left(\hat{P}-P\right)\right)dt+K*u_{\text{f}}$$

## 4 Simulation analysis and experimental verification

### 4.1 Network verification

In the exploration of applying neural networks to solve the FKP, we initially collected the requisite datasets in accordance with established approaches. Subsequently, we employed BP neural network technology to train the model systematically, with the parameters of the joint motor serving as the input of the network and the coordinate position of the robot arm end as the output target.

**Figure 8:**
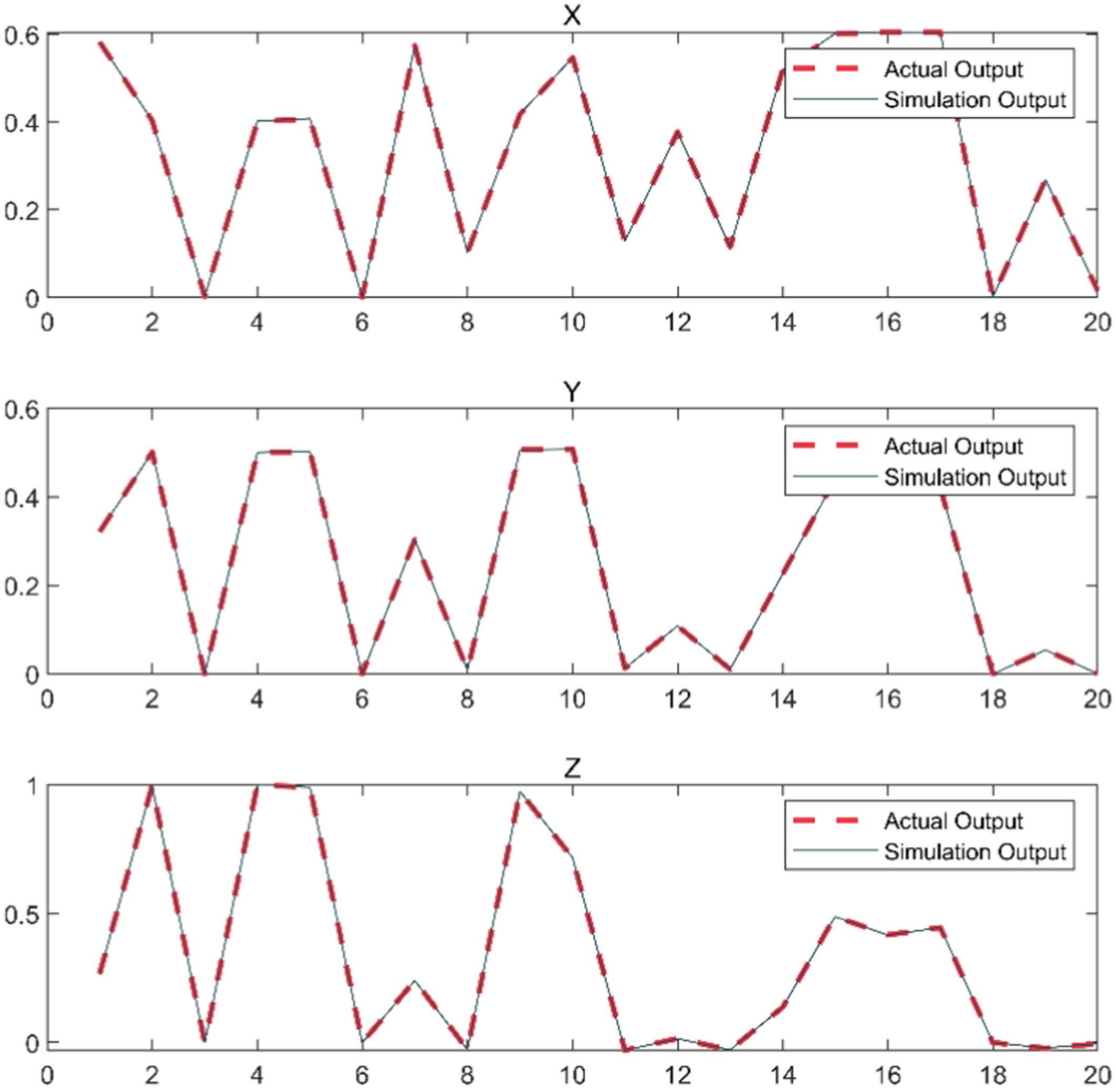
Comparison of end coordinate prediction results.

Once the model training was accomplished, we randomly chose some data samples that were not encompassed in the training to test the prediction capability of the neural network. Based on the outcomes presented in Figure 8, we discovered that the neural network could precisely predict the coordinate position of the robot arm end. This finding thoroughly substantiates that the approach of leveraging the BP neural network to predict the position of the robot arm end is not only feasible but also highly accurate.

Additionally, this data-driven prediction method possesses stronger adaptability and flexibility compared to the traditional analytical method, and can handle the complex scenarios in practical applications more effectively.

### 4.2 Kinematics verification

Based on the characteristic parameters of the robotic arm links and the end effector spherical wrist, the correctness of both forward and inverse kinematics algorithms can be verified through curve acquisition in the driver software. The planner generates the trajectory curve of the robot end effector, and joint values are obtained through inverse kinematics. Then, spatial positions are calculated through forward kinematics, plotting numerous position points into a spatial curve. The degree of overlap between the two curves is compared.

**Figure 9:**
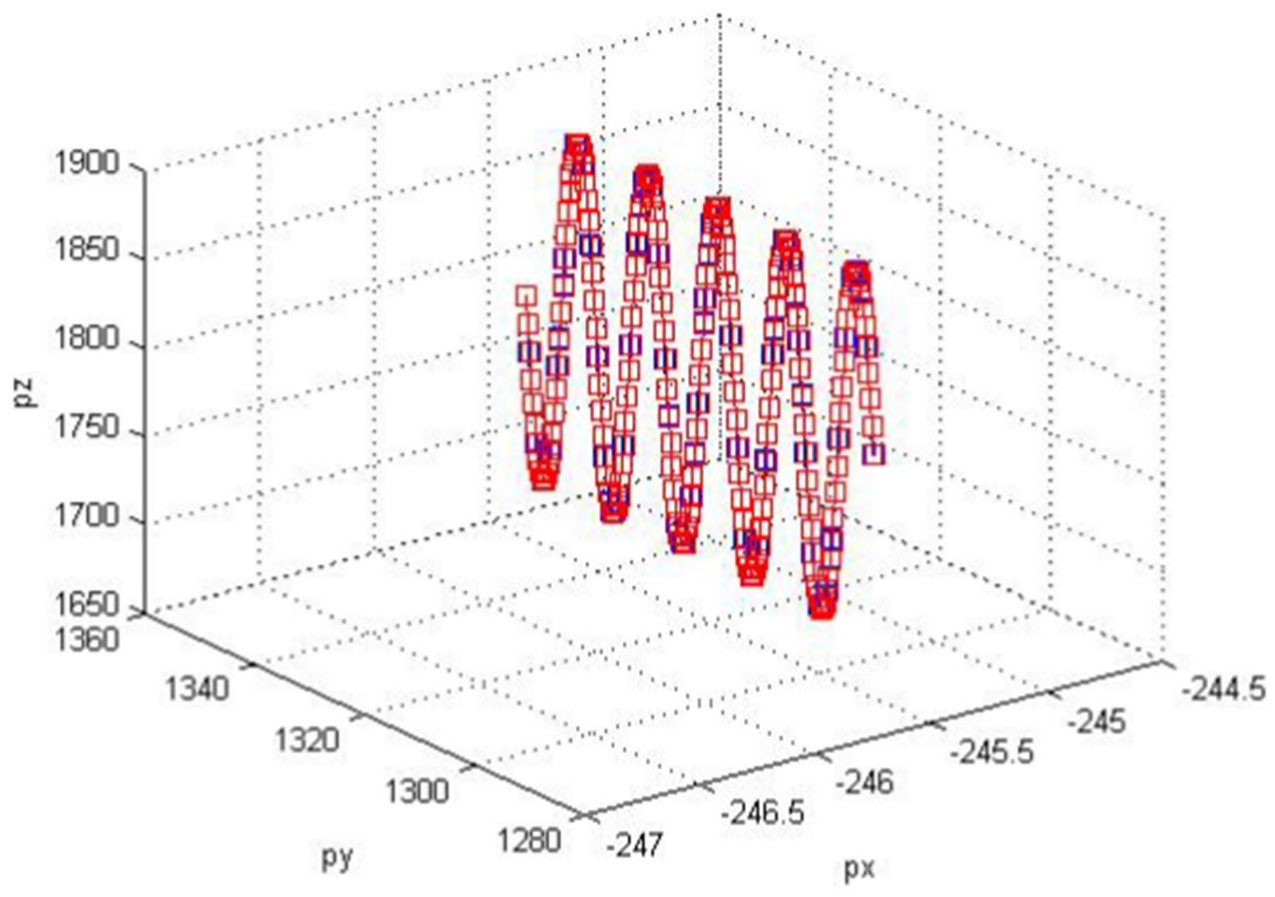
Sine tracking curve.

**Figure 10:**
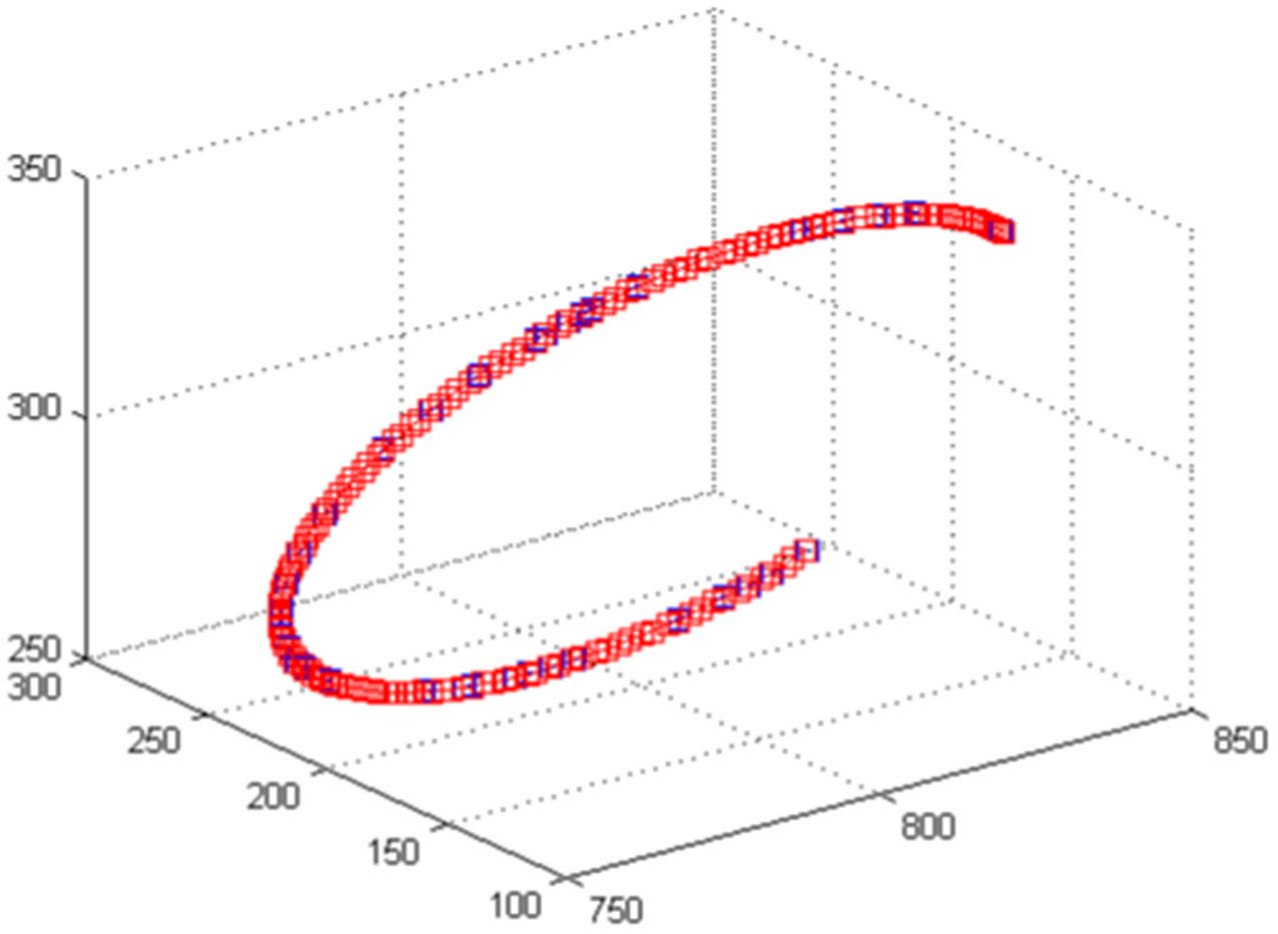
Circular arc trace curve.

Sinusoidal trajectories and spatial circular arc trajectories are separately applied to track trajectories using kinematic algorithms. As illustrated in Figures 9, 10, these are curves collected by the driver software.

### 4.3 Trajectory planning verification

**Figure 11:**
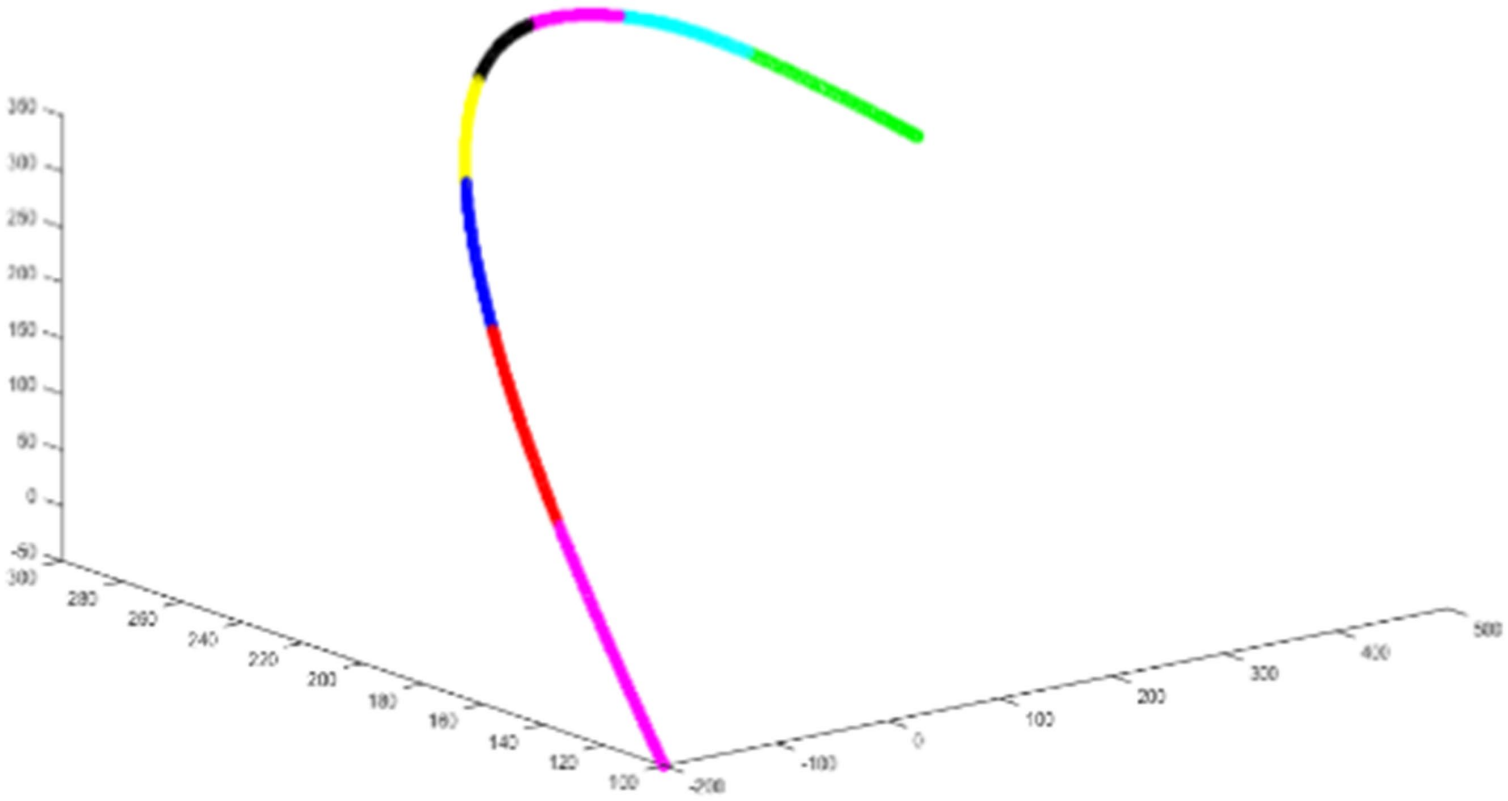
NURBS spline diagram.

The NURBS curve functions as a generalized parameter interpolator, maintaining a uniform feed rate for most of the interpolation process and ensuring that each interpolation point falls within a specified error range. As shown in Figure 11, the interpolator avoids sharp corners and feed-sensitive angles in the curve, thereby mitigating high-frequency components or frequencies matching the machine inherent frequency in the interpolation trajectory, reducing high jitter. In the trajectory planning method, a forward-looking module detects sharp corners of the NURBS curve. An acceleration-deceleration method then adjusts the feed rate at these corners to meet error requirements and accommodate the robot acceleration and deceleration capabilities.

### 4.4 Collision detection algorithm

**Figure 12:**
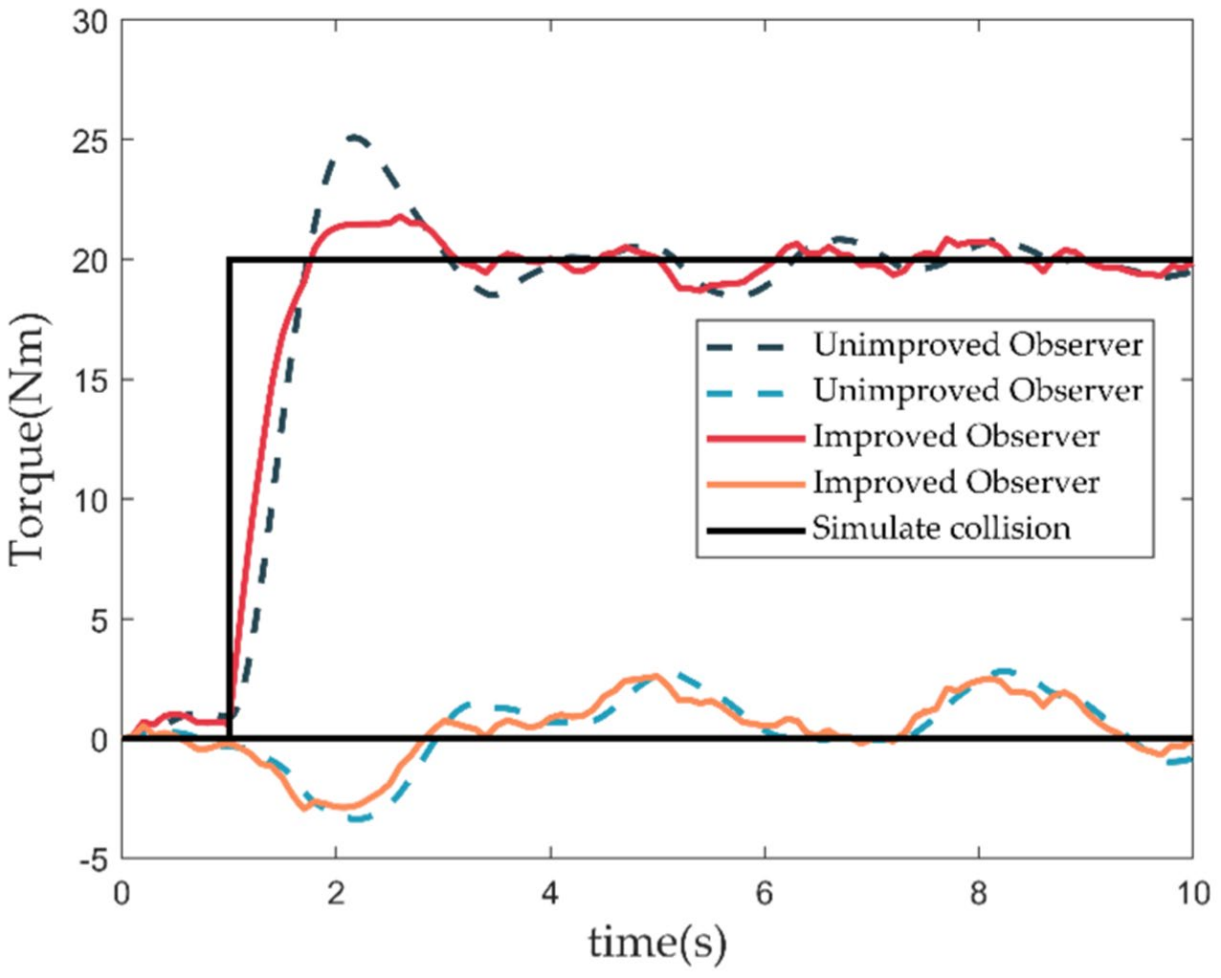
Collision detection simulation.

To quickly verify the feasibility of the collision detection algorithm, we conducted simulation experiments using a two-degree freedom robot model for comparative research. As shown in Figure 12, the dashed line represents the simulation results of the unimproved observer (Equation 18). In contrast, the solid red and green lines represent the simulation results of the improved torque observer (Equation 20). The solid black line simulates the occurrence of a collision. It can be observed that after the collision occurs, the improved observer exhibits reduced overshoot and quick response speed.

### 4.5 True machine verification

**Figure 13:**
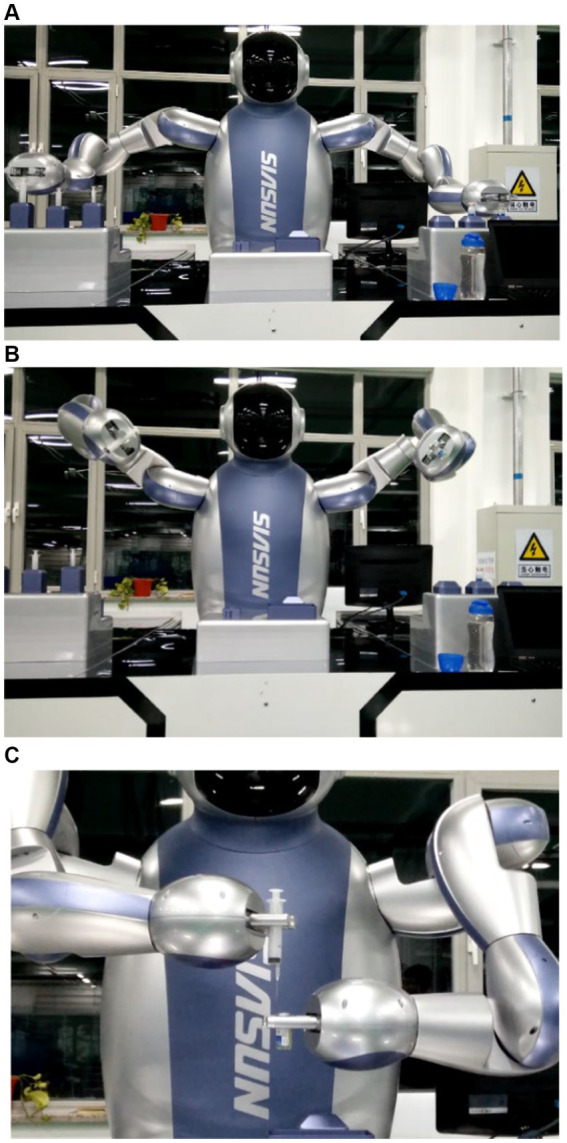
**(A)** Medical robot medicine dispensing. **(B)** Medical robot medicine dispensing. **(C)** Medical robot medicine dispensing.

This paper conducts experiments on flexible control and trajectory planning using the medical dual-arm collaborative robot produced by Siasun Corporation. Figure 13A depicts the experimental results of drug dispensing by the medical assistance dual-arm robot.

(1) Opening the bottle cap: (demonstrated using preopened medication, placing the opened aluminum cap into a box)

- The robot left arm grabs the bottle while the right arm grasps the bottle opener.
- The left arm places the bottle in the specified position and visually checks the notch position. If the position is incorrect, the left arm rotates the bottle to a specific position. Subsequently, the right arm grabs the bottle opener and quickly presses down to remove the aluminum cap from the medication using pressure.
- The right arm returns the bottle opener to its original position, while the left arm lifts the bottle and places the pressed aluminum cap into the collection box. A visual system is introduced here to confirm whether the bottle cap has been successfully opened. An error message is prompted if it is still attached to the bottle or the opener.
- After disposing of the aluminum cap, the bottle is placed back in the specified position.

(2) Medication dispensing demonstration:

- The right arm grabs the syringe, as shown in Figure 13B, while the left grabs the plunger.
- The two arms cooperate to demonstrate liquid suction from the vial and inject the liquid into the bottle, as shown in Figure 13C.

## 5 Conclusion

This paper addresses the challenges of flexible control and trajectory planning in medical dual-arm collaborative robots. It proposes a neural network based kinematic solver as a universal approximator to resolve the kinematic problems of humanoid dual-arm robots while ensuring solution accuracy. Compared to other numerical and geometric methods for solving equation systems, the neural network based kinematic solver directly obtains reasonable solutions in the workspace when the number of unknowns exceeds the number of equations, providing a concise selection approach. Regarding trajectory planning, we propose a comprehensive interpolation scheme based on NURBS curve interpolation. The NURBS spline fitting method is employed to further smooth the interpolated feed curves, with repeated checks for chord error during interpolation to restrict it within a specified error range. Additionally, an improved second-order momentum torque observer is designed to accurately detect external collisions without external sensors. This observer operates without requiring arm acceleration as input, effectively avoiding interference and errors caused by acceleration. Optimized observer design significantly reduces system overshoot, thereby enhancing collision detection accuracy.

## Data Availability

The data supporting this study’s findings are available from the corresponding author upon reasonable request.
